# Preoperative Anemia and Coronary Artery Disease as Predictors of Major Adverse Cardiac Events After Open Abdominal Aortic Surgery

**DOI:** 10.3390/jcm15020738

**Published:** 2026-01-16

**Authors:** Jovan Petrovic, Slobodan Pesic, Natasa Davidovac, Djurdjija Jelicic, Smiljana Stojanovic, Mihailo Neskovic, Bojan Vucurevic, Petar Dabic, Petar Otasevic, Dragana Unic-Stojanovic, Slobodan Tanaskovic, Milovan Bojic

**Affiliations:** 1 Institute for Cardiovascular Diseases Dedinje, 11040 Belgrade, Serbia; 2 General Hospital Cuprija, 35230 Cuprija, Serbia; 3 Faculty of Medicine, University of Belgrade, 11000 Belgrade, Serbia

**Keywords:** coronary artery disease, abdominal aortic aneurysm, anemia, hemoglobin

## Abstract

**Background/Objectives**: Coronary artery disease (CAD) is highly prevalent in patients undergoing vascular surgery and is a major determinant of postoperative morbidity and mortality. Preoperative anemia is a well-recognized risk factor for adverse outcomes, including major adverse cardiac events (MACEs), but its independent impact in patients with CAD undergoing abdominal aortic aneurysm (AAA) repair remains unclear. **Methods**: We conducted a retrospective cohort study of 410 consecutive patients undergoing open AAA repair at a tertiary vascular center between 2023 and 2025. Preoperative anemia was defined as hemoglobin < 130 g/L and significant CAD as ≥70% luminal narrowing for non-left main disease or ≥50% for left main disease. The primary outcome was MACE (cardiovascular death, myocardial infarction, or stroke) during hospitalization. Baseline covariates included age, sex, diabetes mellitus (DM), chronic kidney disease (CKD), congestive heart failure (CHF), peripheral artery disease (PAD), and other relevant comorbidities. Multivariable logistic regression models were used to evaluate associations of anemia, CAD, and their interaction with MACE. Additionally, a composite risk group was created to examine MACE rates across mutually exclusive subgroups. **Results**: Among 410 patients, 314 (76.6%) had CAD and 116 (28.3%) had preoperative anemia. Overall, 67 patients (16.3%) experienced MACE. In the reduced model including only anemia and CAD, anemia remained a strong independent predictor of a MACE (OR 4.46, 95% CI 2.57–7.72, *p* < 0.001), and CAD was also independently associated (OR 2.20, 95% CI 1.00–4.72, *p* = 0.044). In the full multivariable model adjusting for DM, CHF, CKD, PAD, and age, anemia was the strongest predictor (OR 4.53, 95% CI 2.49–8.26, *p* < 0.001), while CAD showed a borderline association (OR 2.07, 95% CI 0.94–4.57, *p* = 0.071). Interaction analysis indicated no statistically significant modification in risk by the combination of anemia and CAD. The composite risk group variable was omitted due to collinearity with its components. **Conclusions**: Preoperative anemia, particularly in patients with CAD, is a significant and independent predictor of major adverse cardiac events following open AAA repair. These findings support the importance of early identification and correction of anemia before surgery to improve perioperative cardiac outcomes in this high-risk population.

## 1. Introduction

Coronary artery disease (CAD) is highly prevalent among patients undergoing vascular surgery and represents the leading cause of postoperative morbidity and mortality in this population [1,2]. Patients with coexisting CAD are at particularly high risk of major adverse cardiac events (MACEs), with reported rates of up to 17% following open aortic repair and associated mortality exceeding 5% [1,3]. Nevertheless, current European Society for Vascular Surgery guidelines do not recommend routine preoperative cardiac evaluation in patients undergoing AAA repair, a strategy that may contribute to unfavorable outcomes in this heterogeneous and high-risk population [4]

Perioperative anemia is a well-established risk factor for adverse outcomes in major vascular surgery, including open abdominal aortic aneurysm (AAA) repair [5,6]. Preoperative anemia has been linked to prolonged hospitalization, increased intensive care unit admissions, and a greater requirement for red blood cell transfusions. Despite its clinical relevance, anemia remains underdiagnosed and undertreated in a substantial proportion of surgical patients [6,7]. Timely preoperative identification and correction of anemia are therefore essential components of perioperative optimization and may significantly improve surgical outcomes [8].

This study aims to investigate the impact of preoperative anemia in patients with CAD undergoing AAA surgery.

## 2. Materials and Methods

This study was a retrospective analysis of prospectively collected data derived from an institutional vascular surgery registry. The registry includes consecutive patients undergoing vascular procedures at a tertiary referral vascular center. Patients who underwent open AAA repair between January 2023 and October 2025 were eligible for inclusion. Data collected before May 2025 were analyzed retrospectively, while from May 2025 onward, data were collected prospectively according to a predefined protocol following institutional ethical approval.

A total of 410 consecutive patients undergoing elective open AAA repair were included. Eligibility criteria included the availability of complete preoperative hematological and cardiovascular assessments. Patients with missing key variables required for analysis were excluded (n = 113). No significant difference in key variables was found between excluded and included patients. The study population consisted of asymptomatic patients with AAA treated electively with major open surgery, defined as procedures associated with an estimated blood loss > 500 mL or operative duration exceeding 2 h [9,10,11].

Two primary preoperative risk factors were evaluated. Significant CAD was defined as angiographically or clinically estimated luminal stenosis ≥ 70% in non–left main coronary arteries or ≥50% in the left main coronary artery [1]. Preoperative anemia was defined as a hemoglobin concentration < 130 g/L based on the most recent hemoglobin measurement within 6 months before surgery [9,10,11,12]. This time window was selected to maximize cohort inclusion in this retrospective analysis, as preoperative laboratory assessments in elective AAA patients are frequently performed several weeks to months before surgery in routine clinical practice.

Baseline covariates included age, sex, hypertension (HTA), diabetes mellitus (DM), chronic kidney disease (CKD), smoking status (smoker vs. non-smoker), heart failure (HF), chronic obstructive pulmonary disease (COPD), atrial fibrillation (AF), and peripheral artery disease (PAD). These variables were selected a priori based on established clinical relevance and previous literature.

The primary outcome was the occurrence of a MACE, defined as a composite of cardiovascular death, myocardial infarction (MI), or stroke during the index hospitalization.

### Statistical Analysis

Data were analyzed by parametric and nonparametric methods. Observed characteristics were expressed as absolute and relative numbers, mean values, standard deviation, or median and interquartile range (IQR). The Kolmogorov–Smirnov test was used to assess the assumption of normality. The Mann–Whitney U test was used for continuous nonparametric data, and Student’s *t*-test was used for parametric data. Categorical data were analyzed using the Chi-square test and Fisher’s exact test to determine the statistically significant difference.

Furthermore, three analytical strategies were also used to evaluate the role of CAD and preoperative anemia in perioperative cardiac risk. The association between preoperative anemia and MACE was assessed using multivariable logistic regression. CAD and other clinically relevant covariates were included in the model as potential confounders. Adjusted odds ratios (ORs) with 95% CIs were reported. Second, to explore whether the effect of anemia differed between patients with and without CAD, an interaction term was added: MACE ∼ anemia + CAD + (anemia × CAD) + covariates. A significant interaction would suggest that the combination of CAD and anemia confers risk beyond the sum of their individual effects. Because some subgroup cell sizes were small, the interaction results were considered exploratory and interpreted descriptively. Finally, four mutually exclusive groups were created for descriptive purposes: non-CAD and no anemia; CAD only; anemia only; and CAD with anemia. MACE rates were reported for each category. Because event numbers were limited, these comparisons were presented descriptively rather than as formal hypothesis tests. A Cochran–Armitage trend test was performed to evaluate whether increasing cardiovascular burden (from neither condition to CAD + anemia) was associated with higher MACE incidence. A multivariable logistic regression model with the composite group as a categorical variable (reference: neither condition) was also fitted to estimate adjusted ORs.

Significance was set at a 2-sided *p* < 0.05. IBM SPSS Statistics v. 26 (Armonk, New York, NY, USA) was used for the analysis. The number of MACEs was limited, reducing statistical power, particularly for interaction and subgroup analyses. Therefore, emphasis was placed on effect sizes and confidence intervals rather than solely on *p*-values.

## 3. Results

Demographic parameters and risk factors are listed in Table 1. Out of 410 patients included, 314 (76.6%) had significant CAD. A total of 116 (28.3%) patients had reported anemia before surgery, with no significant difference between the CAD and non-CAD groups (29.0% vs. 26.0%, *p* = 0.576). The median revised cardiac risk index (RCRI) for the cohort was 2 (IQR 1), and the median VSG-CRI was 5 (IQR 2). Median length of stay (LOS) was 13 (IQR 5) days. Infrarenal clamping was used in the majority of cases (94.2%). Transrenal clamp was utilized in 5 (2.4%) patients, suprarenal clamp in 6 (2.9%), and supracoelica clamp in only one patient (0.5%). Given the small number of patients undergoing non-infrarenal clamping, clamp level was not associated with postoperative outcomes.

In the entire cohort, 67 (16.3%) patients had MACE during hospitalization: 10 cardiovascular deaths (2.4%), and 57 MIs (13.9%). No CVI were observed; so, the composite MACE included only MI and cardiovascular death. In 3 (0.7%) patients, major amputation was performed as a surgical complication. Risk factors related to the primary outcome are given in Table 2. Patients with MACE had a significantly higher incidence of preoperative anemia (56.7% vs. 22.7%, *p* < 0.001) as well as CAD (86.6% vs. 74.6%, *p* = 0.035). Other risk factors which were more prevalent in the MACE group were HF (47.8% vs. 34.7%, *p* = 0.043) and dialysis (3.0% vs. 0.0%, *p* = 0.026). Median RCRI did not differ significantly between groups, while median VSG-CRI score was significantly higher in MACE patients.

To isolate the independent associations of the two primary exposures, a reduced logistic regression model was fitted, including only preoperative anemia and coronary artery disease as predictors of a MACE. In this model, preoperative anemia remained a strong and statistically significant predictor (OR 4.46, 95% CI 2.57–7.72, *p* < 0.001). CAD was also independently associated with an increased risk of MACEs, reaching statistical significance (OR 2.20, 95% CI 1.00–4.72, *p* = 0.044). Both variables contributed meaningfully to the prediction of adverse postoperative events.

In Table 3, a summary of logistic regression models is shown. In the multivariable logistic regression model including preoperative anemia, CAD, DM, HF, CKD, PAD and age, preoperative anemia was the strongest independent predictor of MACEs (OR 4.53; 95% CI: 2.49–8.26; *p* < 0.001). Patients with anemia had more than a fourfold increased likelihood of experiencing a perioperative cardiovascular event compared with non-anemic patients. CAD demonstrated a clinically meaningful but statistically non-significant association with MACE (OR 2.07, 95% CI: 0.94–4.57, *p* = 0.071). HF showed a similar non-significant trend toward increased risk (OR 1.57; 95% CI: 0.90–2.75; *p* = 0.112). DM (OR 1.52, *p* = 0.180), CKD (OR 0.92, *p* = 0.860), PAD (OR 1.47, *p* = 0.245) and age (OR 1.005, *p* = 0.850) were not independently associated with postoperative MACE.

In the multivariable logistic regression model that included the interaction term between anemia and CAD, none of the predictors reached statistical significance. Anemia showed an increased odds of the outcome (OR 2.138, 95% CI 0.513–8.907), but this association was not statistically significant (*p* = 0.297). CAD was also not a significant predictor (OR 1.342, 95% CI 0.484–3.724, *p* = 0.572). The anemia × CAD interaction term did not demonstrate a significant modifying effect (OR 2.477, 95% CI 0.518–11.833, *p* = 0.256), indicating no evidence of effect modification. Given the limited number of events, interaction analyses were considered exploratory and interpreted cautiously. Among the other covariates, DM (OR 1.567, *p* = 0.156), CHF (OR 1.582, *p* = 0.109), PAD (OR 1.531, *p* = 0.202) and CKD (OR 0.813, *p* = 0.813) were not independently associated with the outcome. Age also showed no significant effect (OR 1.003, *p* = 0.906). Overall, the interaction model did not identify any statistically significant predictors, suggesting that when the interaction between anemia and CAD is taken into account, no variable independently predicts the outcome in this dataset.

Absolute rates of perioperative MACE are summarized in Table 4. Patients with both CAD and preoperative anemia (risk group 3) exhibited the highest crude incidence of MACEs (37.4%), while the lowest rates were observed in patients with neither condition (7.0%). Patients with CAD only or anemia only had intermediate event rates (16.0% and 10.8%, respectively), supporting an additive rather than synergistic effect of these risk factors.

The composite risk group variable was entered into the multivariable logistic regression model together with its individual components (preoperative anemia, CAD, DM, HF, CKD, PAD and age). In the final model, preoperative anemia remained an independent predictor of the outcome, with an OR of 4.53 (95% CI 2.49–8.26, *p* < 0.001). CAD did not show association (OR 2.07, 95% CI 0.94–4.57, *p* = 0.071). DM, HF, CKD, PAD and age were not significant predictors (all *p* > 0.10).

## 4. Discussion

The findings of this study demonstrate that preoperative anemia is associated with an increased risk of MACEs following open AAA repair. Patients with anemia experienced substantially higher rates of perioperative MACE, and this association was most pronounced in those with coexisting CAD. In both the full multivariable model and the reduced model including only anemia and CAD, preoperative anemia was consistently associated with more than a fourfold increase in the odds of perioperative MACE, highlighting its clinical relevance as a risk marker in this high-risk surgical population. In contrast, CAD demonstrated a trend toward increased risk in the fully adjusted model and reached statistical significance only in the reduced model, suggesting that anemia may capture perioperative cardiac vulnerability not fully explained by the presence of CAD alone.

Systematic reviews have consistently linked low preoperative hemoglobin with increased mortality and major ischemic events in patients undergoing vascular and other major surgical procedures, even after adjusting for comorbidities and procedural complexity [5]. Observational studies in similar vascular cohorts have further shown that lower preoperative hematocrit levels are independently associated with higher rates of MACEs and mortality, with the strongest effects observed in those with more severe anemia [13]. These findings align with the international Patient Blood Management consensus, which emphasizes the importance of diagnosing and correcting anemia before surgery [9].

Preoperative anemia in our cohort was the strongest and most consistent predictor of MACEs, with adjusted odds ratios of 4.53 in the full multivariable model (Analysis A) and 4.46 in the reduced model including only anemia and CAD. These results are consistent with evidence indicating that hemoglobin levels reflect cardiovascular reserve and tolerance to surgical stress, particularly in patients with CAD. Anemia reduces oxygen delivery, promotes myocardial ischemia, and can trigger sympathetic overactivation, all of which contribute to perioperative cardiac complications [10]. The timing and chronicity of anemia appear critical: pre-existing deficits in oxygen-carrying capacity may predispose patients to ischemic injury during intraoperative hemodynamic fluctuations, especially in those with CAD [14]. These observations support consideration of hemoglobin optimization as part of broader preoperative risk mitigation strategies while acknowledging that causality cannot be inferred from the present study [8].

Large-scale studies corroborate these findings. Musallam et al., analyzing over 227,000 non-cardiac surgery patients, reported that even mild anemia (hematocrit 29–39%) increased 30-day mortality by 42% and overall morbidity by 35% (pooled ORs ~1.4) [7]. Similarly, Li et al. found in a multi-institutional study of infrarenal AAA repair that preoperative anemia, present in 22–25% of patients, was independently associated with a 2- to 3-fold increase in 30-day adverse outcomes, including bleeding, cardiac events, readmission, and non-home discharge (aOR 1.39–3.69; *p* < 0.001) [15]. Major abdominal surgery, including AAA repair, is often complicated with massive perioperative and postoperative bleeding, which can lead to complications such as hemodynamic instability, need for blood cell transfusion, prolonged hospital stay and many more. Patients with preoperative anemia have lower tolerance to bleeding; so, it is clear that they are more likely to experience perioperative and postoperative complications and be at higher risk for MACE than those with normal preoperative hemoglobin levels [16].

Importantly, although patients with both anemia and CAD experienced the highest absolute rates of MACEs, no statistically significant anemia × CAD interaction was observed, indicating an additive rather than synergistic effect. This suggests that anemia and CAD contribute independently to perioperative cardiac risk, reflecting the cumulative burden of cardiovascular vulnerability rather than effect modification. Several perioperative factors—including blood loss, transfusion, and intraoperative hemodynamic instability—may further influence postoperative cardiac outcomes and potentially mediate the observed associations. While these variables could not be incorporated into multivariable models due to incomplete and non-standardized recording, their clinical relevance is acknowledged and represents an important area for future prospective investigation.

The observed association between preoperative anemia and increased risk of MACEs, particularly in patients with CAD, may be explained by the chronic physiological impact of anemia on myocardial oxygen supply. Preoperative anemia reflects a sustained reduction in oxygen-carrying capacity, which can predispose patients, especially those with limited coronary reserve, to myocardial ischemia during periods of surgical stress and hemodynamic instability [17]. Moreover, in patients with CAD, the mismatch between oxygen supply and demand is already pronounced; the presence of preoperative anemia may exacerbate this imbalance, increasing the risk of cardiac complications. Preoperative anemia also frequently coexists with comorbidities such as malnutrition, renal dysfunction, and systemic inflammation, all of which contribute to poorer outcomes [18]. Accordingly, preoperative anemia likely functions both as a potentially modifiable risk factor and as a marker of underlying systemic illness. Although no interaction effect was detected, the combined presence of anemia and CAD was associated with progressively higher MACE rates, consistent with an additive risk framework. The absence of a statistically significant interaction may be partly attributable to limited statistical power, particularly given the number of events.

Biomarkers of myocardial injury and wall stress, particularly high-sensitivity (hs) troponin T and N-terminal pro–B-type natriuretic peptide (NT-proBNP), have been shown to correlate with the severity of coronary artery disease, the presence of anemia, and adverse cardiovascular outcomes across multiple clinical settings [19]. Elevated levels of these biomarkers reflect underlying myocardial vulnerability and subclinical ischemia, which may be exacerbated by reduced oxygen-carrying capacity in anemic patients. Although hs troponin T and NT-proBNP were not routinely assessed in all patients in the present retrospective cohort and therefore could not be included in the current analyses, their established prognostic value suggests that their systematic preoperative measurement may provide additional risk stratification beyond clinical variables alone. Future prospective studies integrating hemoglobin levels with cardiac biomarkers may help clarify the link between anemia, CAD burden, and perioperative myocardial injury, and improve identification of patients at highest risk for adverse outcomes.

An important clinical implication of these findings is the potential value of preoperative anemia screening and optimization as part of comprehensive risk stratification for major vascular procedures. Preoperative anemia is frequently underdiagnosed and undertreated, yet early correction through iron supplementation, erythropoiesis-stimulating agents, or targeted management may mitigate postoperative complications and reduce the burden of MACEs. Recent perioperative care guidelines have recognized the adverse impact of anemia and emphasize patient blood management approaches to improve outcomes, including optimization of hemoglobin before elective surgery [20]. While CAD is a well-established risk factor for perioperative cardiac events, recent evidence suggests that rigorous preoperative optimization, including identification and medical management of underlying coronary disease, can reduce perioperative MACE. Retrospective studies in vascular surgery patients with CAD have observed that thorough cardiovascular assessment and management can influence perioperative outcomes, highlighting the importance of individualized preoperative evaluation [21].

### Limitations

This study has several limitations. First, as an observational study, it can identify associations but cannot establish causality, and there remains the possibility of residual confounding from unmeasured factors such as nutritional status or intraoperative variables. Estimated blood loss, transfusion, operative time, and intraoperative hemodynamic instability were not consistently recorded in a standardized manner across the study period and therefore could not be reliably incorporated into multivariable models. Second, the relatively small sample size, particularly in subgroups like patients with both CAD and anemia, may have reduced the statistical power to detect significant differences, especially regarding postoperative anemia. Additionally, the timing and definition of anemia were based on available hemoglobin measurements, which may vary between patients and influence classification. This study also did not standardize anemia management strategies, such as iron supplementation or transfusion, which could have impacted outcomes. Finally, the follow-up period focused on short- to mid-term MACE, potentially missing longer-term complications, and subclinical myocardial injury (MINS) was not assessed, which may underestimate the true cardiac risk associated with anemia [22,23].

## 5. Conclusions

Preoperative anemia is associated with an increased risk of major adverse cardiac events following open AAA repair, particularly in patients with coexisting CAD. The findings suggest an additive burden of cardiovascular risk rather than a synergistic interaction. Identification of anemia as part of preoperative risk stratification may help refine perioperative cardiovascular assessment in this high-risk population, while the potential benefit of anemia correction requires confirmation in prospective studies.

## Figures and Tables

**Table 1:** Demographic and clinical characteristics of patients.

Factor	Total N = 410
Male sex (n, %)	364 (88.8)
Age [years] (median, IQR)	70 (9)
BMI [kg/m^2^] (median, IQR)	26.3 (5.1)
Preoperative anemia (n, %)	116 (28.3)
Previous MI (n, %)	154 (37.6)
AF (n, %)	64 (15.6)
HF (n, %)	151 (36.8)
HLP (n, %)	357 (87.1)
HTA (n, %)	392 (95.6)
DM (n, %)	97 (23.7)
Smoking (n, %)	289 (70.5)
CKD (n, %)	37 (9.0)
Dialysis (n, %)	2 (0.6)
COPD (n, %)	24 (5.9)
PAD (n, %)	97 (23.7)
RCRI (median, IQR)	2 (1)
VSG-CRI (median, IQR)	5 (2)
LOS [days] (median, IQR)	13 (5)

IQR—Interquartile range, BMI—body mass index, MI—myocardial infarction, AF—atrial fibrillation, HF—heart failure, HLP—hyperlipoproteinemia, HTA—hypertension, DM—diabetes mellitus, CKD—chronic kidney disease, COPD—chronic obstructive pulmonary disease, PAD—peripheral arterial disease, RCRI—revised cardiac risk index, VSG-CRI—Vascular Study Group of New England Cardiac Risk Index, LOS—length of stay.

**Table 2:** Variables according to the primary outcome.

Variables	MACE N = 67	No-MACE N = 343	*p*
Male sex (n, %)	59 (88.1)	305 (88.9)	0.838
Age [years] (median, IQR)	72 (10)	70 (9)	0.048
BMI [kg/m^2^] (median, IQR)	26.0 (4.5)	26.4 (5.2)	0.180
CAD (n, %)	58 (86.6)	256 (74.6)	0.035
Preoperative anemia (n, %)	38 (56.7)	78 (22.7)	<0.001
Previous MI (n, %)	26 (38.8)	128 (37.3)	0.818
AF (n, %)	6 (9.0)	58 (16.9)	0.101
HF (n, %)	32 (47.8)	119 (34.7)	0.043
HLP (n, %)	63 (94.0)	294 (85.7)	0.064
HTA (n, %)	66 (98.5)	326 (95.0)	0.329
DM (n, %)	21 (31.3)	76 (22.2)	0.106
Smoking (n, %)	46 (68.7)	243 (70.8)	0.351
CKD (n, %)	8 (11.9)	29 (8.5)	0.362
Dialysis (n, %)	2 (3.0)	0 (0.0)	0.026
COPD (n, %)	4 (6.0)	20 (5.8)	1.000
PAD (n, %)	19 (28.4)	78 (22.7)	0.322
RCRI (median, IQR)	2 (2)	2 (1)	0.268
VSG-CRI (median, IQR)	5 (2)	5 (2)	0.026
LOS [days] (median, IQR)	16 (7)	13 (5)	<0.001

IQR—Interquartile range, BMI—body mass index, CAD—coronary artery disease, MI—myocardial infarction, AF—atrial fibrillation, HF—heart failure, HLP—hyperlipoproteinemia, HTA—hypertension, DM—diabetes mellitus, CKD—chronic kidney disease, COPD—chronic obstructive pulmonary disease, PAD—peripheral arterial disease, RCRI—revised cardiac risk index, VSG-CRI—Vascular Study Group of New England Cardiac Risk Index, LOS—length of stay.

**Table 3:** Summary of logistic regression models.

Variables	Analysis A OR (95% CI), *p*	Analysis B OR (95% CI), *p*	Analysis C OR (95% CI), *p*
Anemia	4.532 (2.486–8.260), <0.001	2.138 (0.513–8.907), 0.297	4.532 (2.486–8.260), <0.001
CAD	2.073 (0.941–4.568), 0.071	1.342 (0.484–3.724), 0.572	2.073 (0.941–4.568), 0.071
CAD × Anemia	/	2.477 (0.518–11.833), 0.256	/
DM	1.524 (0.824–2.822), 0.180	1.567 (0.843–2.915), 0.156	1.524 (0.824–2.822), 0.180
CHF	1.574 (0.900–2.754), 0.112	1.582 (0.902–2.774), 0.109	1.574 (0.900–2.754), 0.112
CKD	0.922 (0.374–2.276), 0.860	0.813 (0.361–2.226), 0.813	0.922 (0.374–2.276), 0.860
Age	1.005 (0.959–1.053), 0.850	1.003 (0.957–1.051), 0.906	1.005 (0.959–1.053), 0.850

Analysis A: Full multivariable logistic regression model including anemia, CAD, DM, CHF, CKD, and age. Analysis B: Includes the interaction term between CAD and anemia. No predictors reached statistical significance. Analysis C: Composite risk group variable was omitted due to perfect collinearity with its components; the final model is identical to Analysis A. OR—odds ratio; CI—confidence interval; CAD—coronary artery disease, DM—diabetes mellitus; HF—heart failure; CKD—chronic kidney disease.

**Table 4:** Absolute MACEs by Risk Group.

Risk Group	Definition	Patients (n)	MACEs (n)	MACE Rate (%)
0	No CAD, No anemia	71	5	7.0
1	CAD only	25	4	16.0
2	Anemia only	223	24	10.8
3	CAD + Anemia	91	34	37.4
Total	-	410	67	16.3

CAD—coronary artery disease; MACEs—major adverse cardiac events.

## Data Availability

The data presented in this study are available on request from the corresponding author due to ethical reasons.

## Abbreviations

The following abbreviations are used in this manuscript:

CAD	Coronary Artery Disease
MACEs	Major Adverse Cardiac Events
AAA	Abdominal Aortic Aneurysm
DM	Diabetes Mellitus
CKD	Chronic Kidney Disease
HF	Heart Failure
PAD	Peripheral Artery Disease
LOS	Length of Stay
